# Achieving treatment goals of reducing or maintaining body iron burden with deferasirox in patients with β-thalassaemia: results from the ESCALATOR study

**DOI:** 10.1111/j.1600-0609.2011.01661.x

**Published:** 2011-10

**Authors:** Ali Taher, Mohsen S Elalfy, Kusai Al Zir, Shahina Daar, Abdullah Al Jefri, Dany Habr, Ulrike Kriemler-Krahn, Bernard Roubert, Amal El-Beshlawy

**Affiliations:** 1 American University of Beirut Medical CenterBeirut, Lebanon; 2 Ain Shams UniversityCairo, Egypt; 3 National Thalassemia CenterDamascus, Syrian Arab Republic; 4 Sultan Qaboos UniversityMuscat, Oman; 5 King Faisal Specialist Hospital and Research CenterRiyadh, Saudi Arabia; 6 Novartis PharmaceuticalsEast Hanover, NJ, USA; 7 Novartis Pharma AGBasel, Switzerland; 8 Cairo UniversityCairo, Egypt

**Keywords:** deferasirox, iron chelation therapy, iron overload, β-thalassaemia, efficacy

## Abstract

This analysis evaluated the effects of deferasirox on liver iron concentration in moderate and heavily iron-overloaded patients with β-thalassaemia from the ESCALATOR trial (*n* = 231). Mean liver iron concentrations (LIC) decreased significantly from 21.1 ± 8.2 to 14.2 ± 12.1 mg Fe/g dry weight (dw) at 2 yr (*P* < 0.001) in patients with LIC ≥7 mg Fe/g dw at baseline; patients with LIC <7 mg Fe/g dw maintained these levels over the treatment period. The proportion of patients with LIC <7 mg Fe/g dw increased from 9.4% at core baseline to 39.3% by the end of year 2. The results showed that deferasirox enabled therapeutic goals to be achieved, by maintaining LIC in patients with LIC <7 mg Fe/g dw at a mean dose of 22.4 ± 5.2 mg/kg/d and significantly reducing LIC in patients with LIC ≥7 mg Fe/g dw at a mean dose of 25.7 ± 4.2 mg/kg/d, along with a manageable safety profile.

Transfusion therapy in the treatment of patients with β-thalassaemia leads to the development of iron overload and subsequent tissue and organ damage if patients are not chelated effectively. Patients with a liver iron concentration (LIC) >7 mg Fe/g dry weight (dw) are at an increased risk of progressive organ dysfunction and early death as a result of iron-related complications (1). The clinical sequelae of transfusional iron overload may include myocardial disease, liver fibrosis, perturbed endocrine function and retarded growth (1). It is therefore important to reduce LIC below this threshold in patients with LIC ≥7 mg Fe/g dw and maintain levels in patients with LIC <7 mg Fe/g dw, with effective iron chelation therapy. This predefined analysis from the prospective, multicentre ESCALATOR trial (2) evaluates the 2-yr efficacy and safety of the once-daily oral iron chelator deferasirox in patients with LIC <7 or ≥7 mg Fe/g dw at enrolment. This manuscript partners the detailed results from 2.7 yr of the ESCALATOR study (3).

## Methods

### Study design and deferasirox dosing

The ESCALATOR trial was a prospective, open-label, multicentre study conducted in seven sites in the Middle East (2); data reported in this paper are based on patients from six sites. The seventh site was excluded for a good clinical practice reason. The ESCALATOR study design and inclusion/exclusion criteria have been described in full previously (2). The trial consisted of a 1-yr core study, after which patients were eligible to enter into a 2-yr extension study. In the core study, patients started on deferasirox 20 mg/kg/d except for three patients who received an initial dose of 10 mg/kg/d, which was subsequently increased to 20 mg/kg/d based on protocol amendment. Dose adjustments by 5/10 mg/kg/d were performed based on serum ferritin trends and safety markers during the core and extension. Dose increases to >30 mg/kg/d were permitted in the extension study (range 0–40 mg/kg/d).

### Assessments

Efficacy was assessed based on change from core baseline in LIC and serum ferritin levels after 2 yr of treatment (i.e. 1 yr core + year 1 of extension). Efficacy is not presented for year 2 of the extension because of the observed decline in the number of patients remaining on study during this period, once deferasirox became available commercially. (Such patients were noted as study completers but were removed from study assessments after transferring to commercially available deferasirox.) Patients with a core baseline LIC of ≥7 mgFe/g dw had a therapeutic goal of LIC reduction, while in patients with a baseline LIC of 2 to <7 mg Fe/g dw, the therapeutic goal was maintenance. LIC was determined by biopsy at core baseline and after 1 yr and by magnetic resonance imaging after 2 yr. Serum ferritin levels were assessed at baseline and every 4 wk. Safety and tolerability were evaluated across the entire study period by monitoring the incidence and type of adverse events (AEs) and by assessing routine laboratory parameters. Left ventricular ejection fraction (LVEF) measurements by echocardiogram scan were also recorded. Proteinuria was assessed using second void morning urine and defined as above an upper limit of normal (ULN) of 0.149 g/L.

### Statistical methods

Statistical methods used were as described previously for the core study (2). Only patients who enrolled for the extension study are included in this analysis. The efficacy analyses are presented for the intent-to-treat population, which includes all patients who enrolled into the extension study, irrespective of whether they received study drug during the extension. The safety population for which safety data are analysed includes all patients who entered the extension study and received at least one dose of deferasirox.

## Results

### Patient characteristics and dose adjustments

Of 233 patients who completed the core study (2), all were enrolled to continue into the extension study and 231 received at least one dose of deferasirox during the extension. Two patients entered the extension study but did not receive deferasirox and had LIC ≥7 mg Fe/g dw at core baseline. Of the patients who received at least one deferasirox dose during the extension study, 22 had LIC <7 mg Fe/g dw and 209 had LIC ≥7 mg Fe/g dw at core baseline (Table 1). One patient who had a LIC value <7 mg Fe/g dw at baseline did not undergo liver biopsy at the end of the core study.

**Table 1:** Demographics at core baseline of patients who received at least one deferasirox dose in the extension study

	Baseline LIC <7 mg Fe/g dw (*n* = 22)	Baseline LIC ≥7 mg Fe/g dw (*n* = 209)
Mean age ± SD, yr	9.4 ± 4.3	13.4 ± 7.1
Patients aged 2 to <16 yr, *n* (%)	20 (90.9)	142 (67.9)
Patients aged ≥16 yr, *n* (%)	2 (9.1)	67 (32.1)
Female/male, *n*	9 : 13	105 : 104
History of hepatitis B and/or C, *n* (%)	2 (9.1)	69 (33.0)
Splenectomy, *n* (%)	4 (18.2)	89 (42.6)
Previous chelation therapy, *n* (%)		
DFO monotherapy	20 (90.9)	164 (78.5)
Deferiprone monotherapy	0	4 (1.9)
DFO + deferiprone	2 (9.1)	41 (19.6)
Median duration of previous chelation therapy (range), yr^1^	6.0 (0.3–15.6)	6.7 (0.1–21.0)
Mean number of transfusion sessions in the year prior to study entry ± SD	15.2 ± 3.1	15.1 ± 4.4
Mean amount transfused in the year prior to study entry ± SD, mL/kg	165 ± 42	173 ± 70
Mean baseline LIC ± SD, mg Fe/g dw	4.5 ± 1.8	21.1 ± 8.2^2^
Median baseline serum ferritin (range), ng/mL	1921 (914–5696)	3621 (998–25 008)^2^

1 Patients with LIC <7 mg Fe/g dw *n* = 16, patients with LIC ≥7 mg Fe/g dw *n* = 154.

2 *n* = 211.

SD, standard deviation; DFO, deferoxamine; LIC, liver iron concentrations.

### Deferasirox dosing and iron intake

During the core and extension, mean deferasirox dose in the LIC <7 and ≥7 mg Fe/g dw cohorts was 22.4 ± 5.2 and 25.7 ± 4.2 mg/kg/d over median treatment periods of 123.0 and 143.4 wk, respectively. Sixteen patients (72.7%) with baseline LIC <7 mg Fe/g dw and 190 patients (90.9%) with baseline LIC ≥7 mg Fe/g dw had dose increases during the study. The median time to first dose increase was 32 wk (range 8–101) in the LIC <7 mg Fe/g dw cohort and 39 wk (range 3–132) in the ≥7 mg Fe/g dw cohort. The numbers of patients who had dose decreases (one of 22 [4.5%]; 10 of 209 [4.8%]) and dose interruptions owing to AEs and laboratory abnormalities (two of 22 [9.1%]; 26 of 209 [12.4%]) were comparable among the LIC <7 and ≥7 mg Fe/g dw cohorts, respectively. By the end of the extension study, two patients (9.1%) in the LIC <7 mg Fe/g dw cohort and 54 patients (25.8%) in the LIC ≥7 mg Fe/g dw cohort were receiving 40 mg/kg/d deferasirox. The final doses received by patients in the LIC <7 and ≥7 mg Fe/g dw cohorts are detailed in Table 2. Mean iron intake during the core and extension studies was also comparable in the LIC <7 (0.34 ± 0.07 mg/kg/d) and ≥7 mg Fe/g dw (0.33 ± 0.10 mg/kg/d) cohorts.

**Table 2:** Final deferasirox dosing for patients who received at least one deferasirox dose in the extension study

Patient final dose in mg/kg/d, *n* (%)	Baseline LIC <7 mg Fe/g dw (*n* = 22)	Baseline LIC ≥7 mg Fe/g dw (*n* = 209)
5	0 (0)	0 (0)
10	0 (0)	1 (0.5)
15	1 (4.5)	3 (1.4)
20	6 (27.3)	28 (13.4)
25	3 (13.6)	20 (9.6)
30	4 (18.2)	56 (26.8)
35	4 (18.2)	33 (15.8)
40	2 (9.1)	54 (25.8)
Prematurely discontinued	2 (9.1)	14 (6.7)

LIC, liver iron concentrations.

### Efficacy

**Figure 1:**
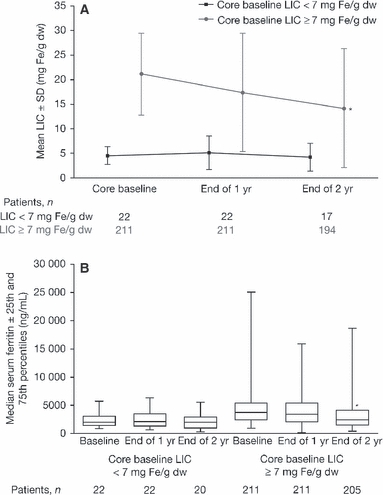
(A) Mean liver iron concentrations (LIC) ± SD and (B) median serum ferritin ± 25th/75th percentiles during deferasirox treatment in all patients enrolled in the extension study, by LIC at core baseline. Whiskers represent minimum and maximum values. *P < 0.001 at end of 2 yr vs. core baseline.

**Figure 2:**
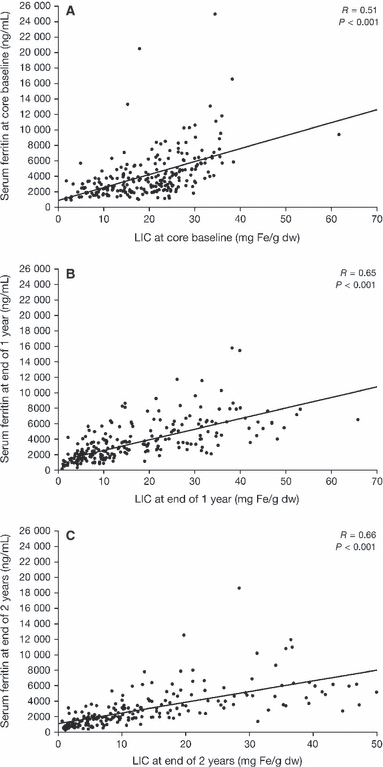
Scatter plot of serum ferritin and liver iron concentrations at (A) core baseline; (B) end of 1 yr; and (C) end of 2 yr.

In the LIC <7 mg Fe/g dw cohort, mean LIC remained stable (mean change of −0.2 ± 3.0 mg Fe/g dw; Fig. 1A) over 2 yr (core baseline: 4.5 ± 1.8 mg Fe/g dw; at 2 yr 4.3 ± 2.7 mg Fe/g dw). In the LIC ≥7 mg Fe/g dw cohort, mean LIC decreased significantly from 21.1 ± 8.2 mg Fe/g dw at core baseline to 14.2 ± 12.1 mg Fe/g dw at 2 yr (overall mean change −7.2 ± 9.6 mg Fe/g dw, *P* < 0.001; Fig. 1A). The proportion of patients with LIC <7 mg Fe/g dw increased from 22/233 (9.4%) at core baseline to 61/233 (26.2%) at the end of 1 yr and to 83/211 (39.3%) at the end of year 2. A similar pattern of response was observed with serum ferritin levels (Fig. 1B). A summary of the number of patients with LIC <7, 7 to <10 and ≥10 mg Fe/g dw at core baseline, at the end of 1 yr and at the end of 2 yr, by baseline LIC category, is shown in Table 3. Median serum ferritin levels were maintained around core baseline levels (1921 ng/mL) in the LIC <7 mg Fe/g dw cohort (1915 ng/mL after 2 yr, median change −145 ng/mL; *P* = 0.418) and decreased significantly in the LIC ≥7 mg Fe/g dw cohort (3621 ng/mL at core baseline, 2398 ng/mL after 2 yr, median change −1052 ng/mL; *P* < 0.0001). A significant correlation between serum ferritin and LIC was observed at core baseline (*r* = 0.51; *P* < 0.001), at the end of 1 yr (*r* = 0.65; *P* < 0.001) and at the end of 2 yr (*r* = 0.66; *P* < 0.001, Fig. 2). In order to investigate whether any baseline characteristic could be used to identify patients with end-of-study (EOS) LIC <7 or ≥7 mg Fe/g dw, univariate analysis of baseline characteristics were performed. Only LIC and serum ferritin were significantly different (*P* < 0.0001 for both) at baseline in patients with EOS LIC <7 or ≥7 mg Fe/g dw (Table 4).

**Table 3:** Patients with LIC <7, 7 to <10 and ≥10 mg Fe/g dw at core baseline, at the end of 1 yr and at the end of 2 yr, by baseline LIC category

	LIC category, mg Fe/g dw [*n* (%)]			
		
Baseline LIC	<7 mgFe/g dw	7 to <10 mgFe/g dw	≥10 mgFe/g dw	Total
*Core baseline*				
<7 mg Fe/g dw	21 (95.5)	1 (4.5)^1^	0 (0.0)	22 (9.4)
≥7 mg Fe/g dw	2 (0.9)^1^	18 (8.5)	191 (90.5)	211 (90.6)
Total	23 (9.9)	19 (8.2)	191 (82.0)	233 (100.0)
*End of year 1*				
<7 mg Fe/g dw	18 (81.8)	3 (13.6)	1 (4.5)	22 (9.4)
≥7 mg Fe/g dw	43 (20.4)	27 (12.8)	141 (66.8)	211 (90.6)
Total	61 (26.2)	30 (12.9)	142 (60.9)	233 (100.0)
*End of year 2*				
<7 mg Fe/g dw	15 (88.2)	1 (5.9)	1 (5.9)	17 (8.1)
≥7 mg Fe/g dw	68 (35.1)	25 (12.9)	101 (52.1)	194 (91.9)
Total	83 (39.3)	26 (12.3)	102 (48.3)	211 (100.0)

LIC, liver iron concentrations; MRI, magnetic resonance imaging.

1 Baseline LIC categories were assessed using liver biopsy. LIC categories during the study were assessed by MRI. This resulted in the classification of one patient with a baseline LIC <7 mg Fe/g dw with an LIC category of 7 to <10 mg Fe/g dw and classification of two patients with a baseline LIC ≥7 mg Fe/g dw with an LIC category of <7 mg Fe/g dw.

**Table 4:** Analysis of baseline characteristics in patients with EOS LIC <7 mg Fe/g dw or ≥7 mg Fe/g dw

Characteristic at baseline	EOS LIC <7 mg Fe/g dw (*n* = 112)	EOS LIC ≥7 mg Fe/g dw (*n* = 121)	*P*-value
Mean age ± SD, yr	13.7 ± 7.2	12.5 ± 6.7	0.214
Female/male, *n*	54 : 58	60 : 61	0.834
History of hepatitis B and/or C, *n* (%)	28 (25.0)	43 (35.5)	0.081
Splenectomy, *n* (%)	45 (40.2)	50 (41.3)	0.859
Previous chelation therapy, *n* (%)*			0.108
DFO monotherapy	87 (77.7)	99 (81.8)	–
Deferiprone monotherapy	4 (3.6)	0 (0.0)	–
DFO + deferiprone	21 (18.8)	22 (18.2)	–
Median duration of previous chelation therapy (range), yr^1^	7.9 (0.3–21.0)	6.5 (0.1–20.6)	0.228
Mean number of transfusion sessions in the year prior to study entry ± SD^2^	14.6 ± 3.8	15.6 ± 4.6	0.393
Mean amount transfused in the year prior to study entry ± SD, mL/kg^3^	167.1 ± 65.0	175.5 ± 70.4	0.471
Mean baseline LIC ± SD, mg Fe/g dw	14.9 ± 7.9	23.9 ± 8.3	<0.0001
Median baseline serum ferritin (range), ng/mL	2553 (914–7519)	4299 (1154–25 008)	<0.0001

SD, standard deviation; DFO, deferoxamine; EOS, end-of-study; ITT, intent-to-treat; LIC, liver iron concentrations. LIC at EOS is based on last observation carried forward (LOCF) on the ITT population.

* *P*-value calculated for patients receiving previous chelation therapy with any of DFO, deferiprone or DFO/deferiprone.

1 Patients with LIC <7 mg Fe/g dw *n* = 69, patients with LIC ≥7 mg Fe/g dw *n* = 102.

2 Patients with LIC <7 mg Fe/g dw *n* = 101, patients with LIC ≥7 mg Fe/g dw *n* = 118.

3 Patients with LIC <7 mg Fe/g dw *n* = 93, patients with LIC ≥7 mg Fe/g dw *n* = 116.

### Discontinuations and safety

Twenty patients (90.9%) in the baseline LIC <7 mg Fe/g dw cohort and 196 patients (92.9%) in the baseline LIC ≥7 mg Fe/g dw cohort completed the study. Reasons for discontinuation were as follows: loss to follow-up (*n* = 2, 9.1% and *n* = 6, 2.8%), AEs (0% and *n* = 3, 1.4%), death (0% and *n* = 3, 1.4%), protocol violation (0% and *n* = 2, 0.9%) and consent withdrawal (0% and *n* = 1, 0.5%) in the LIC <7 and ≥7 mg Fe/g dw cohorts, respectively. Of the three deaths reported during the study, these were attributed to respiratory failure and as a result of cerebral and subarachnoid haemorrhage; none were considered to be related to study drug. AEs considered by the investigator to be related to deferasirox were reported in 100 patients (43.3%) in the core study. Overall, drug-related AEs were less common in the extension study (55 patients; 23.8%) vs. the core study (100 patients; 43.3%) (Table 5). Serious AEs were reported in two patients (9.1%) in the LIC <7 mg Fe/g dw cohort and 24 patients (11.5%) in the LIC ≥7 mg Fe/g dw cohort. These were considered to be drug-related in three patients, all of whom were in the LIC ≥7 mg Fe/g dw cohort (peptic ulcer perforation, increased serum creatinine levels and cholestatic jaundice).

**Table 5:** Overall and most common investigator-assessed drug-related AEs (≥5% in either cohort) in patients who received at least one deferasirox dose in the extension study, by LIC at core baseline

	Core baseline LIC, mg Fe/g dw			
	
	<7 (*n* = 22)		≥7 (*n* = 209)	
		
Adverse event, *n* (%)	Core	Extension	Core	Extension
Any AE	11 (50.0)	6 (27.3)	89 (42.6)	49 (23.4)
Vomiting	2 (9.1)	2 (9.1)	18 (8.6)	12 (5.7)
Increased serum creatinine	1 (4.5)	1 (4.5)	8 (3.8)	13 (6.2)
Increased ALT	3 (13.6)	3 (13.6)	10 (4.8)	13 (6.2)
Rash	3 (13.6)	0	15 (7.2)	2 (1.0)
Nausea	0	0	14 (6.7)	4 (1.9)
Increased AST	2 (9.1)	0	4 (1.9)	2 (1.0)
Proteinuria	2 (9.1)	0	4 (1.9)	1 (0.5)

ALT, alanine aminotransferase; AST, aspartate aminotransferase; AEs, adverse events; LIC, liver iron concentrations.

LVEF at core baseline was above the lower limit of normal (4) in both LIC cohorts and increased in both during deferasirox treatment. Mean LVEF increased non-significantly from 63.2 ± 6.6% to 67.8 ± 9.5% (mean absolute increase 4.3 ± 13.7%) in the LIC <7 mg Fe/g dw cohort and improved significantly in the LIC ≥7 mg Fe/g dw cohort (65.4 ± 6.8% to 68.3 ± 7.7%; mean absolute increase 2.1 ± 8.1%; *P* = 0.0013). Serum creatinine levels >33% above core baseline and above the ULN were observed in four patients (18.2%) with baseline LIC <7 mg Fe/g dw and in four (1.9%) patients with baseline LIC ≥7 mg Fe/g dw. One patient (4.5%) in the LIC <7 mg Fe/g dw cohort and seven patients (3.3%) in the LIC ≥7 mg Fe/g dw cohort had alanine aminotransferase (ALT) levels >10× ULN on two consecutive occasions; core baseline levels were elevated in one and four patients, respectively. Mean ALT levels decreased in the LIC ≥7 mg Fe/g dw cohort (−27.7 ± 47.4 U/L, *P* < 0.0001; baseline 65.4 ± 46.1 U/L) and remained stable in the LIC <7 mg Fe/g dw cohort (+6.7 ± 47.0 U/L; baseline 35.2 ± 43.2 U/L) at the end of the extension study.

## Discussion

Patients enrolled in the ESCALATOR study were characterised by a very high iron burden, despite receiving prior chelation therapy; most patients (210/233 patients; 90.1%) had LIC of ≥7 mg Fe/g dw at core baseline. Markers of iron overload at core baseline, including serum ferritin levels and LIC (see Table 1), were much greater than levels known to be associated with significant negative outcomes (serum ferritin >2500 ng/mL and LIC >15 mg Fe/g dw) (1, 5) and hence what is generally clinically desirable. This suggests that previous chelation regimens were suboptimal in these patients and were unable to maintain iron levels consistently below these thresholds.

In line with study objectives, treatment with deferasirox over 2 yr was able to maintain iron levels in patients with baseline LIC <7 mg Fe/g dw and to reduce iron load in patients with baseline LIC ≥7 mg Fe/g dw. In the latter cohort, this resulted in a significant reduction in iron burden from core baseline after 2 yr of deferasirox treatment. Given the relatively long median time to first dose increase in both cohorts, it is important to note that more rapid dose escalations may be required in order to achieve therapeutic goals over a shorter time frame. This is particularly important when reduction in iron burden is desired such as in the LIC ≥7 mg Fe/g dw cohort. Although the patient numbers in the LIC <7 mg Fe/g dw cohort are low, the results for both cohorts are consistent with a subanalysis of the large EPIC study in which patients with LIC <7 (*n* = 71) and ≥7 mg Fe/g dw (*n* = 303) experienced a stabilisation or reduction in LIC, respectively, when treated with mean actual deferasirox doses of 20 and 27 mg/kg/d over a 1-yr period (6).

During this study, deferasirox was well tolerated with a clinically manageable safety profile. Most AEs were mild-to-moderate in nature and were consistent with AEs reported in the core trial (2). Mean ALT levels decreased significantly in the LIC ≥7 mg Fe/g dw cohort but remained unchanged in the LIC <7 mg Fe/g dw cohort, reflecting the changes in LIC and serum ferritin levels. Overall, drug-related AEs were less common in the extension study compared with the core study. There was no clear trend in the type or frequency of drug-related AEs between the LIC <7 and ≥7 mg Fe/g dw cohorts.

In conclusion, appropriate deferasirox dosing was able to maintain or reduce iron burden in patients with β-thalassaemia in line with therapeutic goals.
